# Exosomal Linc00969 induces trastuzumab resistance in breast cancer by increasing HER-2 protein expression and mRNA stability by binding to HUR

**DOI:** 10.1186/s13058-023-01720-6

**Published:** 2023-10-17

**Authors:** Cuiwei Liu, Chong Lu, Lamu Yixi, Jiaxing Hong, Fang Dong, Shengnan Ruan, Ting Hu, Xiangwang Zhao

**Affiliations:** 1 Cancer Center, Union Hospital, Tongji Medical College, Huazhong University of Science and Technology, Wuhan, 430022 China; 2 Hubei Key Laboratory of Precision Radiation Oncology, Wuhan, 430022 China; 3 Institute of Radiation Oncology, Union Hospital, Tongji Medical College, Huazhong University of Science and Technology, Wuhan, 430022 China; 4 Department of Breast and Thyroid Surgery, Union Hospital, Tongji Medical College, Huazhong University of Science and Technology, Wuhan, 430022 China; 5 Tibet Shannan Maternal and Child Health Hospital, Shannan, 856000 Tibet China

**Keywords:** Exosomes, Linc00969, Breast cancer, HER-2, HUR

## Abstract

**Background:**

Breast cancer (BC) is the most common malignant disease in female patients worldwide. In HER-2+ BC patients, trastuzumab therapy is associated with a better prognosis. However, many HER-2+ BC patients experience recurrence or metastasis because of trastuzumab resistance. The mechanisms underlying trastuzumab resistance remain unclear. Recently, substantial evidence has suggested that exosomes are associated with drug resistance, and lncRNAs have attracted increasing attention due to their potential role in the regulation of trastuzumab resistance.

**Methods:**

We collected the exosomes from the plasma of BC patients with and without trastuzumab resistance, sequenced the whole transcriptomes, identified differentially expressed lncRNAs, and identified lncRNA Linc00969, which was overexpressed in trastuzumab-resistant patients. Then, we established trastuzumab-resistant BC cell lines and explored the role of exosomal Linc00969 in trastuzumab resistance in vitro and in vivo by silencing or overexpressing Linc00969 and performing a series of functional analyses. Furthermore, to explore the mechanism by which exosomal Linc00969 contributes to trastuzumab resistance, we measured changes in HER-2, HUR and autophagy-related protein expression levels after regulating Linc00969 expression. In addition, we investigated the interaction between Linc00969 and HUR via pull-down and RIP assays and the effect of HUR on HER-2 expression and trastuzumab resistance after blocking HUR.

**Results:**

We first found that exosomal lncRNA Linc00969 was overexpressed in trastuzumab-resistant BC patients and that exosome-mediated Linc00969 transfer could disseminate trastuzumab resistance in BC. Then, we found that silencing Linc00969 could reduce trastuzumab resistance and that overexpressing Linc00969 could enhance trastuzumab resistance*.* Furthermore, our results showed that Linc00969 could upregulate HER-2 expression at the protein level and maintain the stability of HER-2 mRNA by binding to HUR. Additionally, we found that exosomal Linc00969 could regulate trastuzumab resistance by inducing autophagy.

**Conclusions:**

In this study, we first identified that exosomal lncRNA Linc00969 could induce trastuzumab resistance by increasing HER-2 protein expression and mRNA stability by binding to HUR, and Linc00969 might also be involved in trastuzumab resistance by inducing autophagy. Our results elucidate a novel mechanism underlying trastuzumab resistance, and Linc00969 might be a new target for improving the treatment of HER-2+ BC patients.

**Supplementary Information:**

The online version contains supplementary material available at 10.1186/s13058-023-01720-6.

## Background

Breast cancer (BC) is the most common malignant disease in female patients worldwide, and the number of patients is increasing by 0.3% every year [1]. Approximately 15–20% of BC patients are positive for human epithelial growth factor receptor 2 (HER-2) amplification/overexpression. The subtype of these patients is HER-2-enriched BC [2]. Trastuzumab is a recombinant monoclonal antibody that binds to the extracellular domain of HER-2. Trastuzumab is a drug that is recommended for neoadjuvant, adjuvant and advanced first-line treatment in BC patients. It has been proven that it can prolong the survival of HER-2+ BC patients [3, 4]. Although trastuzumab improves the prognosis of HER-2+ BC patients, 25–40% of HER-2+ BC patients still experience recurrence or metastasis due to trastuzumab resistance [2, 5]. Therefore, it is important to clarify the mechanism underlying trastuzumab resistance in HER-2+ BC.

Trastuzumab can inhibit the dimerization of HER-2 protein, suppress the transduction of downstream signals, increase the apoptosis of cancer cells, reduce DNA repair and hinder angiogenesis in HER-2+ BC [6, 7]. Recently, substantial evidence has shown that the abnormal activation of the downstream pathways of HER-2 (PI3K/AKT or RAS/ERK signaling pathway) plays an important role in trastuzumab resistance [8, 9]. A variety of MEK or PI3K inhibitors have been shown to increase trastuzumab sensitivity and reverse trastuzumab resistance in HER-2+ BC patients [10–13]. However, abnormal activation of the PI3K/AKT and RAS/ERK signaling pathways usually coexists and synergistically leads to trastuzumab resistance, and the suppression of a single pathway is not always effective in BC patients resistant to trastuzumab. A large number of studies have shown that the combination of multiple targeted drugs causes powerful damage to HER-2+ BC cells. Many special small molecular compounds, such as BEZ235 (targets both PI3K and mTOR), aim to inhibit multiple signaling pathways to reverse trastuzumab resistance. However, the clinical benefits of the application of these new drugs or the combination of targeted drugs is not clear in trastuzumab-resistant patients, and due to a lack of sufficient efficacy and safety data, most of these new drugs remain in the preclinical stage [14]. Therefore, it is very important to identify new molecules that can target multiple trastuzumab resistance-related signaling pathways.

Exosomes are extracellular vesicles that are 30–100 nm in diameter that contain various biomolecules. Exosomes can be absorbed by recipient cells through endocytosis, phagocytosis and membrane fusion [15], and then the biomolecules carried by exosomes are released into the percipient cells, where the biomolecules can perform various physiological functions [16]. The membrane proteins of exosomes can also bind to membrane proteins of target cells to activate signal pathways in target cells and realize intercellular signal transduction [17]. Tumor cells secrete more exosomes in response to changes in the microenvironment. Substantial evidence suggests that exosome-mediated cell communication is associated with drug resistance [18, 19]. In general, exosomes are involved in tumor drug resistance in two ways: one is that exosomes transfer key drug resistance proteins (such as Rab27B) or RNAs (such as lncRNAs) to tumor cells to induce or enhance drug resistance; the other is that exosomes can phagocytize drug molecules and excrete them outside the cells to reduce the drug concentration in tumor cells [20, 21]. Furthermore, because of the low immunogenicity and high stability of exosomes, they can fuse well with target tumor cells, making exosomes the best choice for drug carriers.

LncRNAs are RNA molecules that are more than 200 nucleotides in length and play a dynamic regulatory role in gene expression and pathology. The abnormal expression of lncRNAs in cancers has shown strong biological effects in regulating proliferation, migration, drug resistance and other malignant behaviors in BC cells [22]. Endogenous lncRNAs can be secreted into body fluids by tumor cells in the form of microbubbles, exosomes or protein complexes, forming stable circulating lncRNAs that are not degraded. Exosomal lncRNAs can spread to recipient cells so that they can cause phenotypic changes in recipient cells. Exosomal lncRNAs can also reprogram tumor cells in the tumor microenvironment and promote tumor development [23].

LncRNAs have attracted increasing attention due to their role in regulating trastuzumab resistance in BC. One recent study showed that lncRNA AFAP1-AS1 can promote HER-2 translation, increase HER-2 expression and cause trastuzumab resistance by binding to AUF1. AFAP1-AS1, which is a lncRNA, can be packaged into exosomes to enhance the trastuzumab resistance of receptor tumor cells [24]. Mechanistically, lncRNA AGAP2-AS1 can increase the acetylation of H3K27 in the MyD88 promoter region, resulting in NF-κB signal pathway activation and trastuzumab resistance [25]. LncRNA SNHG14 has also been reported to regulate acetylation of H3K27 in the PABPC1 gene promoter and induce expression of PABPC1, resulting in Nrf2 signal pathway activation and causing trastuzumab resistance [26]. Another study showed that CBP-mediated acetylation of H3K27 can activate lncRNA TINCR, also leading to trastuzumab resistance [27]. Mechanistically, TINCR can act as a sponge for the miR-125b target HER-2 and upregulate HER-2 expression to decrease the antitumor effect of trastuzumab. However, there is no direct evidence to prove the role of exosomal lncRNAs in trastuzumab resistance in HER-2+ BC patients.

Our research group tried to study whether and how exosomal lncRNAs maintain the activity of the HER-2 signaling pathway and cause trastuzumab resistance. We collected the exosomes from the plasma of patients with and without trastuzumab resistance, sequenced the whole transcriptomes, identified differentially expressed lncRNAs, and identified lncRNA Linc00969, which was overexpressed in trastuzumab-resistant patients and induced BC cell resistance to trastuzumab in vitro.

Based on the above results, we propose that lncRNA Linc00969 may be secreted by being packaging into exosomes and play a role in trastuzumab resistance. To confirm this hypothesis, we established a trastuzumab-resistant BC cell line and performed a series of functional analyses to explore the potential role and mechanism of exosomal Linc00969 in trastuzumab-resistant BC cells. Our results may provide potential therapeutic targets for trastuzumab resistance and facilitate the development of new therapeutic approaches in BC.

## Methods

### Exosomes isolation

Centrifuge the supernatant at 300×*g* 4 °C for 10 min, then harvest the supernatant and centrifuge it at 2000 ×*g* for 10 min, suck the supernatant and centrifuge at 10,000 ×*g* for 30 min, harvest the supernatant and 140,000×*g* overspeed centrifugation for 90 min, remove the supernatant, the sediment is exosomes. Wash the sediment with PBS buffer and centrifuge at 140,000 ×*g* for 90 min, then resuspend the precipitate with 100 μl PBS buffer and stored the samples at − 80 °C until use.

### Patient samples

108 serum samples in total from HER-2+ breast cancer patients who received trastuzumab treatment were collected at Union Hospital, Tongji Medical College, Huazhong University of Science and Technology between June 2015 and June 2018. Samples of 5 ml venous blood from each participant were collected by venipuncture prior to starting trastuzumab treatment. Centrifuge the blood at 1600 × g for 10 min at room temperature within 2 h after collection, then second centrifuge the blood at 12,000 × g for 10 min at 4 °C to remove the residual cells debris. The serum supernatant was transferred into RNase free tubes and stored at -80 °C. All patients were pathologically confirmed, patients with breast benign disease, autoimmune diseases or other types of cancer were excluded.

We evaluated the efficacy of trastuzumab after 2 cycles of treatment. Tumor response was confirmed through computed tomography and evaluated according to the Response Evaluation Criteria In Solid Tumors (RECIST; version 1.1), complete response (CR), partial response (PR), stable disease (SD) and progressive disease (PD). We defined the patients who evaluated with PD were trastuzumab resistant patients, while patients with PR or CR were trastuzumab sensitive patients.

### Expression profile analysis of lncRNAs

The quality control for each sample sequence was carried out by FastQC (http://www.bioinformatics.babraham.ac.uk/projects/fastqc/), the RNA-seq data were compared by using HISAT2 software, and the expression values from experimental group and control group were statistically calculated by DESeq2.0 algorithm. The calculation parameters mainly included: log2FC value, FDR value, P value. The screening criteria for significant difference factors were log2FC > 1 or < − 1, and FDR < 0.05. According to the results of significant difference genes, the cluster diagram was drawn.

### Breast cancer cell lines and cell culture

The human breast cancer cell lines, BT474 and SKBR-3 were acquired from American Type Culture Collection (ATCC) and maintained in McCoy’s 5A with 10% fetal bovine serum and 1% penicillin/streptomycin. The trastuzumab resistant cell lines (BT474-TR and SKBR-3-TR) were established and also maintained in McCoy’s 5A with 10% fetal bovine serum and 1% penicillin/streptomycin. All above cells were cultured at 37 °C with 5% CO_2_ condition.

### Establishment of trastuzumab resistant breast cancer cell lines

Human breast cancer cell lines, SKBR-3 and BT474 cells were treated with trastuzumab (Roche) when the cells grew to 85 ~ 95% density. The initial concentration of trastuzumab is 10 μg/ml, after 24 h induction culture, the cell culture medium was changed to the regular medium without trastuzumab. When the cells grew to 85 ~ 95% density, the cells were stably subcultured for three times at this concentration. Then the breast cancer cells were treated with 20 μg/ml trastuzumab to conduct induction culture for 24 h similar with above steps, untill the cells could stably grow and pass on in the medium at this concentration. Further, the concentration of trastuzumab for induction culture was increased to 40 μg/ml, 60 μg/ml, 80 μg/ml and 100 μg/ml. In this way, we finally obtained trastuzumab resistant breast cancer cells (SKBR-3-TR and BT474-TR) that could stably grow, pass on, cryopreserved and recovered in the culture medium with an effective trastuzumab concentration at 100 μg/ml.

### CCK8 assay

Cell viability was analysed by Cell Counting Kit-8 (CCK8, Beyotime, Shanghai, China) following to the manufacturer's protocols. The human breast cancer cells were seeded and cultured into 96-wells plates. Then, the cells were treated with trastuzumab. Add 10 μL of CCK-8 reagent to each well and culture the samples for 2 h. At last the absorbance was analysed at 450 nm by microplate reader. The wells without cells were treated as blanks.

### Colony forming assays

The breast cancer cells with log phase growth were plated in 6-well plates. The cells were incubated at 37 °C overnight and treated with trastuzumab. Then the cells were fixed with a mixture of methanol and acetic acid (10:1 v/v) and stained with 1% crystal violet in methanol after 10–14 days of incubation in 6-well plates. At last the numbers of colonies with > 50 cells were counted and the surviving fractions were calculated.

### EdU (5-Ethynyl-2-Deoxyuridine) assay

The breast cancer cells were seeded into 96-well plates. The cells were incubated at 37 °C overnight and treated with trastuzumab. Then the cells were incubated with EdU solution for 2 h (1/1000, RiboBio, China). Remove EdU solution and fix the cells with 4% paraformaldehyde for 30 min, permeabilize the cells by 0.5% Triton X-100 for 10 min and stain the cells by Hoechst. At last the EdU positive cells were detected by fluorescent microscope and counted by ImageJ Software.

### Transmission electron microscopy

Centrifuge the breast cancer cells at 1000 rpm, 4 °C for 15 min and collect the cancer cells. Incubate the cells with 2.5% glutaraldehyde solution at 4 °C overnight. Then the cells undergo dehydrating, embedding, solidifying, ultrathin slicing, and staining. At last cell samples were observed and imaged by a transmission electron microscope.

### Quantitative real-time PCR

The RNA extraction was harvested by using TRIzol reagent (Invitrogen). Then the RNA extraction was undergoing reverse transcription by using Prime RT reagent kit (Vazyme). PCR primer sequences (5'to3') are recorded as follows: human Gapdh-F primer sequence CCACATCGCTCAGACACCAT; human Gapdh-R primer sequence TGACAAGCTTCCCGTTCTCA; human Linc00969-F primer sequence ACGGATCACCACTGCAAGAG; human Linc00969-R primer sequence TAGGTGGAATCGGGCCTGTA; human HUR-F primer sequence GAAGACCACATGGCCGAAGA; human HUR-R primer sequence TGGTCACAAAGCCAAACCCT. Quantitative PCR was performed by using SYBR Green real-time PCR kit (Vazyme).

### Western blotting

The whole cell lysates were harvested via cell lysis buffer and the protein concentration was detected with BCA Protein Assay Kit (Thermo). Then the proteins were undergoing separating by 8–12% gradient gels and transferred to PVDF (Polyvinylidene Fluoride) membranes. Membranes were blocked by blocking buffer and incubated with primary antibodies at 4 °C overnight. At last the membranes were incubated with secondary antibodies at room temperature for 1 h and scanned by infrared imaging system. The following primary antibodies were used: TSG101 (1:1000, ab133586, Abcam), CD81 (1:1000, ab109210, Abcam), HUR (1:1000, ab200342, Abcam), HER-2 (1:1000, ab134182, Abcam), GAPDH (1:1000, 60,004–1-Ig, Proteintech), p62 (1:1000, cat. no. 18420–1-AP), LC3 (1:1000, cat. no. 14600–1-AP), CD63 (1:1000, BD Bioscience, clone H5C6), CD9 (1:1000, Millipore, clone MM2/57), Fibronection (1:1200, ab285285, Abcam).

### Immunofluorescence staining

The breast cancer cells were fixed by 4% formaldehyde and underwent permeabilizing by PBS with 0.2% Triton X-100. Then the cells were blocked by blocking buffer and incubated with primary antibody at 4 °C overnight. Finally the cell samples were incubated with secondary antibody for 1 h, washed by PBS, mounted in DAPI (4',6-diamidino-2-phenylindole), and observed under confocal laser scanning fluorescence microscopy.

### RNA interference and overexpression

BT474 and SKBR-3 cells were transfected with Linc00969 overexpression plasmids. BT474-TR and SKBR-3-TR cells were transfected with siRNA-Linc00969. The transfection kit used was riboFECT™ CP kit as directed by manufacturer’s protocols and the breast cancer cells were used in following experiments after 24 h’ transfection. Overexpression or knockdown cells were confirmed by RT-PCR. (siRNA-Linc00969-1: CGAUUCCACCUACAGCAAAGC; siRNA-Linc00969-2: GGACGGAUCACCACUGCAAGA; siRNA-HUR: TCCAGATTTTTGAAAAATACAAT).

### Fluorescence probe in situ hybridization (FISH) assay

Breast cancer cells were fixed by 4% formaldehyde for 20 min, and washed by PBS on a shaker for 5 min × 3 times. Then cells were added protease K (20 μg/ml) to digest for 3 min, and washed by PBS for 5 min × 3 times. Droped the pre-hybridization solution and incubate for 1 h at 37 °C, removed pre-hybridization solution and incubated with probe hybridization solution at 37 °C overnight. Washed the cells by 2 × SSC for 10 min, 1 × SSC for 5 min twice and 0.5 × SSC at 37 °C for 10 min. Finally, cells were mounted in DAPI and observed under fluorescence microscopy.

### In vivo xenograft mouse model

Animal experiments were authorized by Medical Ethics Committee of Union Hospital, Tongji Medical College, Huazhong University of Science and Technology, under national standard guidelines for animal welfare. Nude mice (4 weeks old, 16-18 g, BALB/c Nude) were randomly grouped and five nude mice each group. A suspension of 1–5 × 10^7^ human breast cancer cells in 1 ml PBS was perpared, then the medium was mixed with matrigel at the ratio 1:1 for injection. 1–5 × 10^6^ (100 μl) human breast cancer cells were injected subcutaneously into middle posterior part of axilla of each nude mouse and the mice were treated with trastuzumab. Tumor volume was monitered twice a week and calculated by the formula: V = 1/2 × a × b^2^, where a = length (mm), and b = width (mm).

### Immunohistochemistry staining

The subcutaneous trasnplanted tumores were submitted in cassettes for paraffin embedding and sectioning. The tumor Sects. (4 µm) were incubated with primary antibodies at 4 °C overnight, then underwent incubating with secondary antibody and Streptavidin-Avidin–Biotin. Finally, peroxidase reaction was performed by diaminobenzidine tetrahydrochloride and the sections were counterstained by haematoxylin. The sections were visualized under microscope in five independent high magnification fields.

### Nucleo-cytoplasmic separation

The nuclear and cytosolic samples of breast cancer cells were separated by utilizing PARIS kit (Am1921, Thermo Fisher Scientific, USA) according to the manufacturer’s protocols. The U1, GAPDH and Linc00969 expression levels in nuclear and cytoplasm of breast cancer cells were detected by qRT-PCR.

### RNA pull-down assay

Firstly, the control RNA, target RNA and the probe labeling reaction system were prepared by using Pierce™ RNA 3' End Desthiobiotinylation Kit according to the instructions. The probe labeling reaction system was added into PCR instrument at 16 °C for more than 4 h or overnight, 400 μl nuclease free-water was added into each sample afer reaction. Then 300 μl phenol chloroform was added into samples to extract successfully labeled RNA, centrifuge the samples at fastest speed for 15 min after vibrate, transfer the supernatant to a new EP tube. Secondly, 10 μl 5 M NaCl, 2 μl glycogen and 600 μl pre-colded 100% ethanol were added into the supernatant, then the samples were deposited overnight at -20 °C or -80 °C and centrifuged at fastest speed for 30 min at 4 °C, the precipitate was the RNA sample. Remove the supernatant, wash the RNA by 70% ethanol, centrifuge the samples for 10 min, remove the supernatant again and dry the RNA in air. Finally, 20 μl nuclease free water was added into each sample to dissolve the RNA, then the RNA was added into RNA instrument and denatured for 5 min at 95 °C for following experiments.

The magnetic beads were also need to prepared. Put 400 μl magnetic beads (400 μl for control RNA, 400 μl for target RNA) on the magnetic frame, remove the supernatant, wash the beads by 800 μl 1 × binding & washing buffer 3 times. Then, 400 μl 2 × binding & washing buffer, 20 μl RNA and 380 μl DEPC water were added into the magnetic beads, rotate the samples slowly at room temperature for 20 min, so that the beads could fully bind with RNA. Transfer the samples on the magnetic frame and remove the supernatant, wash the samples by 800 μl 1 × binding & washing buffer 3 times. Finally, the RNA binded beads were washed by cell lysis buffer A for following experiments.

The protein extraction samples were harvested by cell lysis buffer and mixed with the prepared beads (1 U/μl RNase inhibitor was also added), rotate the samples slowly at 4 °C for 2 h, so that the beads could fully combine with protein. Transfer the samples on the magnetic frame, remove the supernatant and wash the samples by 400 μl cell lysis buffer A 5 times. Then, the beads were suspended by 25 μl pre-colded 0.1% SDS solution, added 6.25 μl 5 × protein loading buffer, boiled at 100 °C for 10 min and placed on ice immediately for 5 min. At last the beads were placed on magnetic frame, the supernatant was transfered into a new EP tube for western blotting detection.

### RIP (RNA Binding Protein Immunoprecipitation) assay

Firstly, we should prepare the magnetic beads and antibody. The magnetic beads coated with protein-A/G were fully suspended and washed by NT-2 buffer twice, then the magnetic beads were suspended by 100 μl NT-2 buffer and mixed with 5 μg target antibody in room temperature for 1 h. Centrifuge the magnetic beads at 5000 × g for 15 s, add magnetic base to absorb magnetic beads and remove the supernatant, then use 1 ml NT-2 buffer to wash the magnetic beads 5 times. At last the magnetic beads were suspended by 900 μl NET-2 buffer for following experiments.

The cell lysates were harvested via cell lysis buffer and centrifuged at 4 °C 20000 × g for 10 min. Mix 100 μl cell lysate supernatant with 900 μl NET-2 buffer suspended magnetic beads to carry out antibody incubation. Reserve 10 μl sample for “Input” copy and store it at -80 °C. Mix the other sample by vertical mixer at 4 °C for more than 3 h or overnight. Centrifuge the sample for a short time, and put the sample on the magnetic base upon the ice, remove the supernatant after 1 min at 4 °C, then use 1 ml NT-2 buffer to wash the sample 5 times, the precipitate was the final sample got from RIP assay. The RNA sample could be further extracted after digestion by proteinase K for subsequent analysis.

### Statistics

The values of samples were represented as mean ± SD which measured triply. Comparisons between two groups were analyzed by unpaired Student’s t test or analyzed by ANOVA for experiments that more than 2 subgroups. *P* value was considered statistically significant when it < 0.05. The software Graphpad Prism was utilized for statistical analysis.

## Result

### Isolation and identification of exosomes

Exosomes were isolated from the plasma of trastuzumab-resistant BC patients (R-exo) and trastuzumab-sensitive BC patients (S-exo) and observed by electron microscopy, and the morphology and size conformed to the characteristics of exosomes (Additional file 1: Fig. S1A). Then, we used western blotting to measure the expression of biomarkers of exosomes. We found that R-exo and S-exo both had high levels of proteins (CD63, CD81 and CD9) (Additional file 1: Fig. S1B). The diameter of the exosomes was also determined by particle size identification, and the results confirmed that the diameter of exosomes we derived from patients conformed to the exosome parameters (Additional file 1: Fig. S1C). The exosomes derived from BC cells were also observed by electron microscopy (Additional file 1: Fig. S1D), and the expression of exosome biomarkers (TSG-101, CD81, CD9, CD63 and fibronectin) was extremely high in exosomes derived from BC cells (Additional file 1: Fig. S1E). The diameter and number were also examined by particle size identification, confirming again that we had isolated exosomes (Additional file 1: Fig. S1F). Therefore, we successfully isolated and identified exosomes derived from BC patients and cells.

### Exosomes from trastuzumab-resistant BC cells enhance trastuzumab resistance

**Fig. 1:**
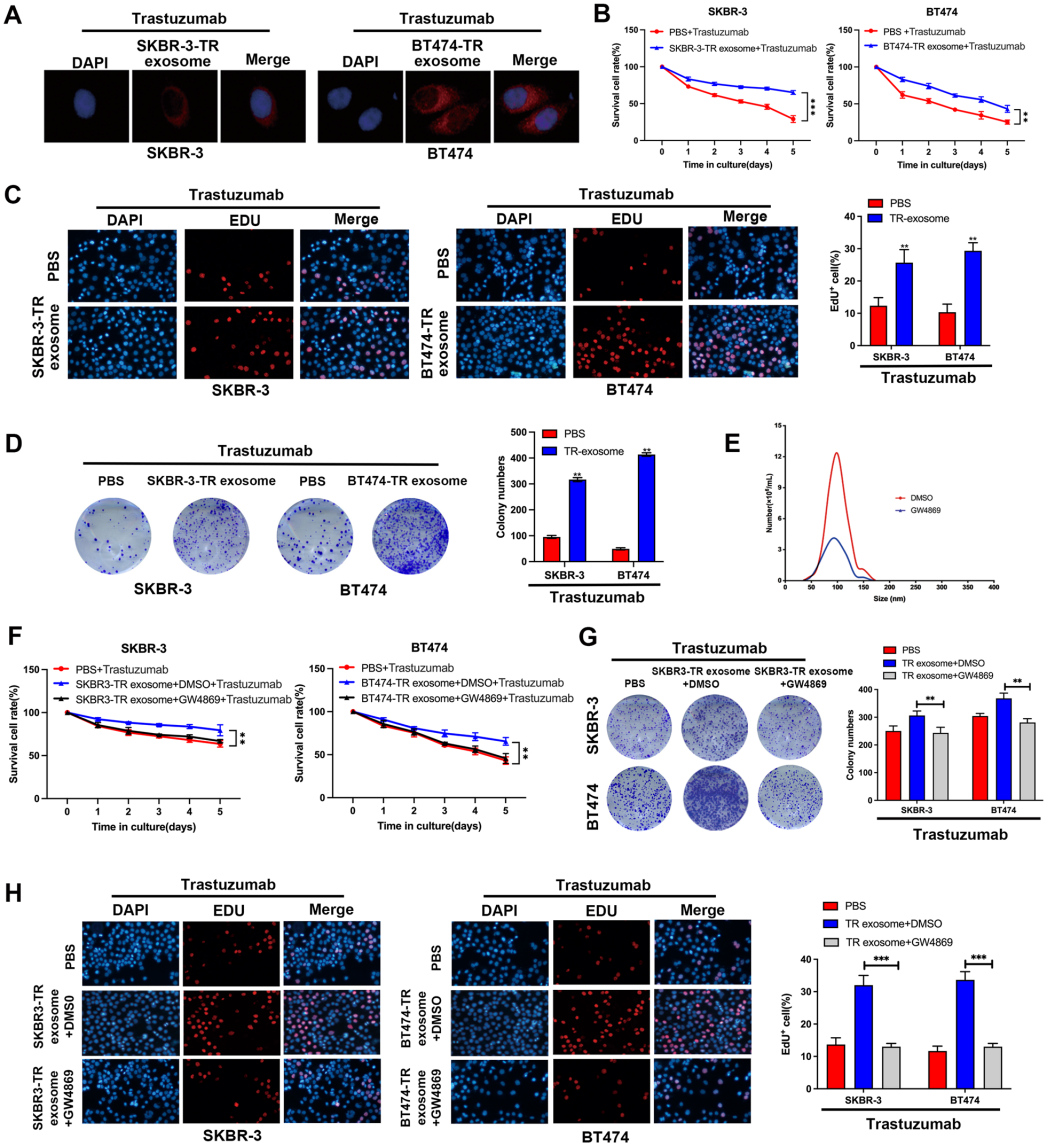
Exosomes from trastuzumab-resistant breast cancer cells enhance trastuzumab resistance. **A** Immunofluorescence staining showed the subcellular localization of exosomes. **B** CCK8 assay. **C** EdU assay. **D** Colony forming assay of breast cancer (BC) cells cultured with or without exosomes derived from trastuzumab-resistant cells after trastuzumab treatment. **P < 0.01. **E** The numbers of secreted exosomes were decreased after treatment with the exosome secretion blocker GW4869. **F** CCK8 assay. **G** Colony forming assay. **H** EdU assay of BC cells with or without GW4869 and exosomes derived from trastuzumab-resistant cells after trastuzumab treatment. **P < 0.01. ***P < 0.001

First, we established trastuzumab-resistant BC cell lines: BT474-TR and SKBR-3-TR (Additional file 1: Fig. S1G). The results showed that exosomes from BT474-TR and SKBR-3-TR cells can be taken up by recipient BC cells within 24 h (Fig. 1A). We further examined whether trastuzumab-resistant cell-derived exosomes could confer trastuzumab resistance in recipient BC cells. Figure 1B shows that parental cancer cells incubated with exosomes derived from BT474-TR or SKBR-3-TR cells exhibited increased viability after trastuzumab treatment. Similar results were also obtained via colony formation assay and EdU assay (Fig. 1C, D). To determine whether exosomes from BC cells were involved in this effect, we reduced exosome production with GW4869 (Fig. 1E). GW4869 is a specific noncompetitive neutral sphingomyelinase (N-SMase) inhibitor with cell permeability. It can block the sprouting of multivesicular bodies mediated by ceramide, thereby inhibiting the biogenesis or release of exosomes. GW4869 is commonly used to inhibit the generation of exosomes [28]. Multiple assays, including CCK8, colony formation and EdU assays, revealed that incubation with culture medium from BT474-TR or SKBR-3-TR cells failed to confer trastuzumab resistance in recipient BC cells after treatment with GW4869 (Fig. 1F–H). The above results proved that trastuzumab-resistant BC cell-derived exosomes can enhance trastuzumab resistance in recipient BC cells.

### Microarray profiling for exosomal lncRNAs involved in trastuzumab resistance

**Fig. 2:**
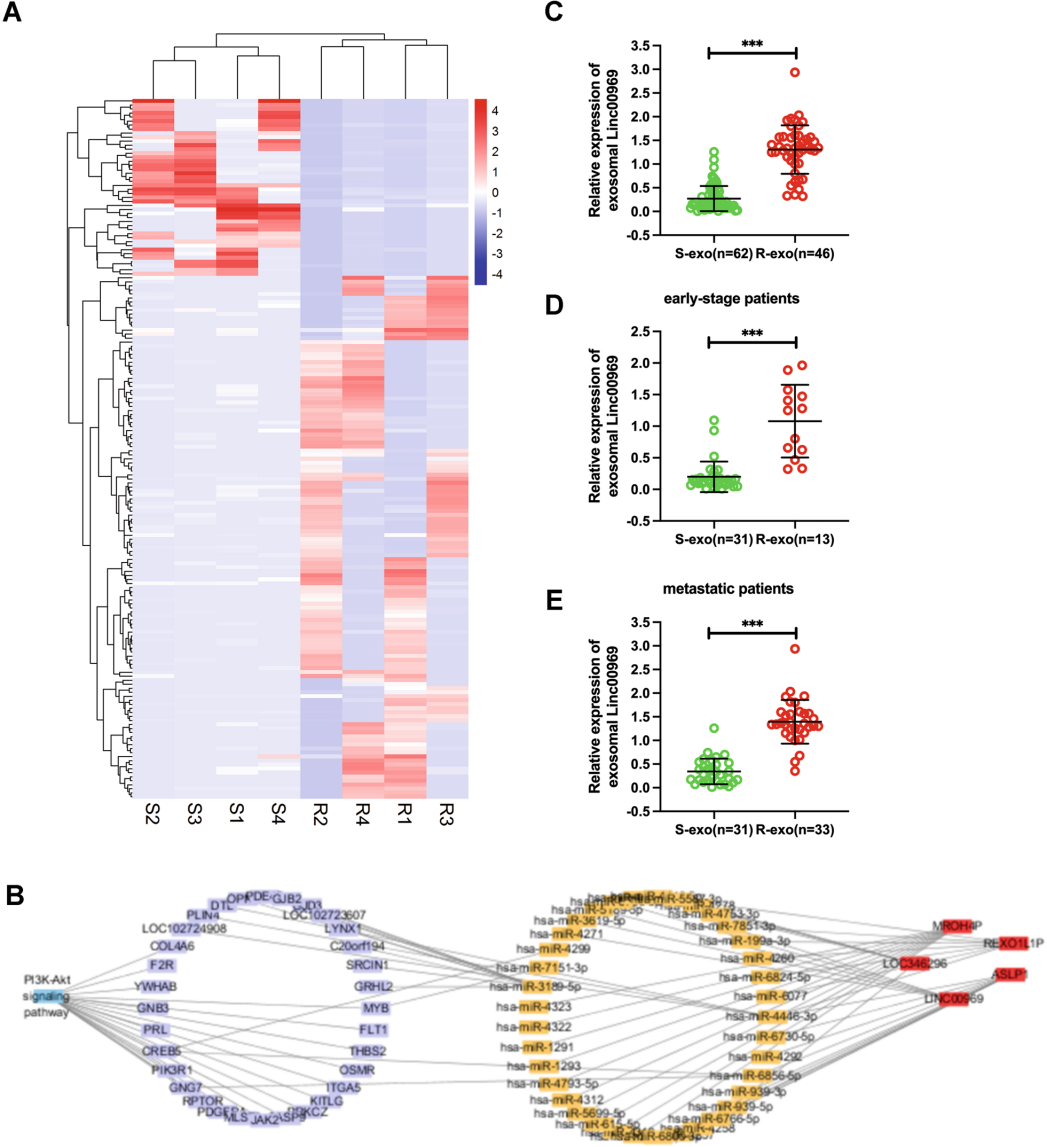
Microarray profiling of exosomal lncRNAs associated with trastuzumab resistance. **A** Heatmap plot showing the cluster analysis of the differentially expressed RNAs (DERs) between plasma exosomes from trastuzumab-resistant (R) breast cancer (BC) patients and trastuzumab-sensitive (S) patients. **B** The ceRNA network analysis indicated the enriched crosstalk based on the altered DERs and PI3K-Akt pathway. **C** qPCR assays validated the overexpressed levels of Linc00969 in plasma exosomes from trastuzumab-resistant BC patients (R-exo) compared with plasma exosomes from trastuzumab-sensitive BC patients (S-exo). ***P < 0.001. **D** Validation of Linc00969 levels in plasma exosomes from early-stage BC patients via qPCR technology. ***P < 0.001. **E** Validation of Linc00969 levels in plasma exosomes from metastatic BC patients via qPCR technology. ***P < 0.001

To identify the underlying mechanism by which exosomes mediate trastuzumab resistance, deep RNA sequencing of exosomal lncRNA and cirRNA were performed, which included samples from four patients sensitive to trastuzumab treatment and samples from four patients resistant to trastuzumab treatment. The sequencing analysis revealed 4785 lncRNAs in total, including 306 upregulated lncRNAs and 4479 downregulated lncRNAs, that were dysregulated in exosomes from trastuzumab-sensitive BC tissues compared with trastuzumab-resistant BC tissues (Additional file 5: Table S1, Fig. 2A). However, we did not find any differences in the expressions of cirRNA (Additional file 6: Table S2). The ceRNA network map of differentially expressed RNAs (DERs) in these transcriptome data was enriched in the PI3K-Akt pathway. Because the PI3K-Akt signaling pathway plays a critical role in the occurrence and development of BC, we screened lncRNA candidates that participate in the PI3K-Akt pathway and were the most upregulated (Fig. 2B). The results showed that the expression trends of MROH4P, REXO1L1P, LOC346296, and LINC00969 were consistent with the sequencing results. LINC00969 was the most significantly different lncRNA found in sequencing test.

Furthermore, we measured the expression levels of the Linc00969 in 108 patients blood samples and 47 tissue samples. We found that linc00969 was significantly increased in trastuzumab-resistant patients blood samples, both in 44 early-stage HER-2+ BC patients and 64 metastatic HER-2+ BC patients (Fig. 2C–E). In total 108 patients blood samples, the patients were divided into high expression group and low expression group based on the median value of Linc00969 expression (0.476) in exosomes. In the trastuzumab sensitive patients (n = 62), the median level of Linc00969 was 0.1267 (SD = 0.8632) and 1.5802 (SD = 0.355) in the trastuzumab resistant patients (n = 36). The Linc00969 expression level in exosome was signifcantly upregulated in the trastuzumab resistant compared with the sensitive patients (*p* < 0.001). Besides, In tissue samples, the median level of Linc00969 was 0.2693 (SD = 0.3102) in the trastuzumab sensitive patients (n = 28) and 1.1430 (SD = 0.9723) in the trastuzumab resistant patients (n = 19). The Linc00969 expression level in tissue was also signifcantly upregulated in the trastuzumab resistant compared with the sensitive patients (*p* < 0.001) (Additional file 1: Fig. S1H).

Additionally, we evaluated the association between exosomal Linc00969 and clinical characteristics in BC. As shown in Additional file 7: Table S3, elevated expression levels of exosomal Linc00969 were significantly associated with positive nodal status, higher histological grade (2/3), higher Ki67 score (≥ 40%) and distant metastasis (*p* < 0.05). The above results showed that high expression level of exosomal Linc00969 in BC patient serum have close correlation with trastuzumab resistance and poor clinical characteristics in BC. Therefore, we focused on Linc00969 in subsequent experiments.

### Exosome-mediated Linc00969 transfer spreads trastuzumab resistance

**Fig. 3:**
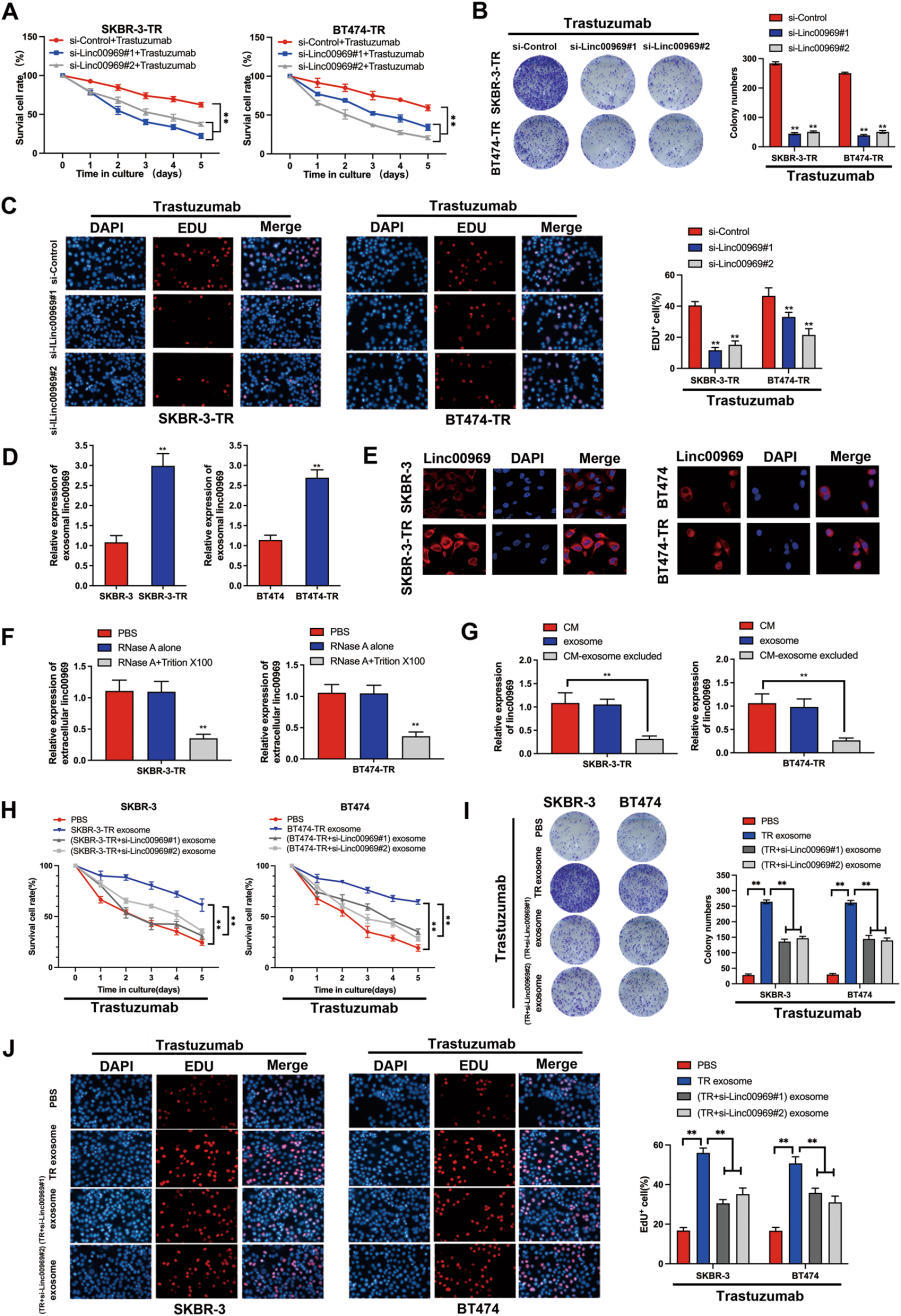
Exosome-mediated transfer of Linc00969 spreads trastuzumab resistance. **A** CCK8 assay. **B** Colony forming assay. **C** EdU assay of breast cancer (BC) cells with or without si-Linc00969 after trastuzumab treatment. **P < 0.01. **D** The expression levels of Linc00969 were measured by PCR in SKBR-3, BT474, BT474-TR and SKBR-3-TR cells. **P < 0.01. **E** FISH assay with a Linc00969 probe showed the subcellular localization of Linc00969 in SKBR-3, BT474, BT474-TR and SKBR-3-TR cells. **F** The expression levels of Linc00969 in exosomes derived from BT474-TR and SKBR-3-TR cells after treatment with RNase A and Triton X100. **P < 0.01. **G** The expression levels of Linc00969 in culture medium and exosomes from BT474-TR and SKBR-3-TR cells.**P < 0.01. **H** CCK8 assay. **I** Colony forming assay. (J) EdU assay of BC cells treated with trastuzumab with or without exosomes derived from trastuzumab-resistant cells or cells treated with si-Linc00969. **P < 0.01

To further investigate whether exosomal Linc00969 was involved in HER-2+ BC trastuzumab resistance, we designed and performed relevant experiments in vitro. First, we stably knocked down Linc00969 expression in trastuzumab-resistant BC cells with siRNA and overexpressed Linc00969 in parental trastuzumab-sensitive cells with plasmids (Additional file 2: Fig. S2A-B). As shown in Fig. 3A-C, CCK8, colony formation and EdU assays indicated that Linc00969 knockdown notably inhibited cell proliferation after trastuzumab treatment, while Linc00969 overexpression obviously increased viability (Additional file 2: Fig. S2C-E). Furthermore, as shown in Fig. 3D, exosomal Linc00969 was markedly upregulated in the trastuzumab-resistant subgroups compared to the parental cell lines. FISH assays with the Linc00969 probe showed that Linc00969 was distributed mainly in the cytoplasm (Fig. 3E). Moreover, the Linc00969 level in the culture medium was nearly unchanged after treatment with RNase alone but sharply decreased after treatment with both RNase and Triton X100. This result indicated that Linc00969 was encapsulated by a membrane instead of being directly released (Fig. 3F). Moreover, the Linc00969 expression levels in exosomes of trastuzumab-resistant cells were almost equivalent to those in culture medium. However, extracellular Linc00969 levels were sharply reduced after removing the exosomes in culture medium (Fig. 3G), which indicated that the exosomes were the main carrier of extracellular Linc00969. More importantly, silencing Linc00969 sharply inhibited the ability of cocultured BC cells to acquire resistance to trastuzumab (Fig. 3H–J). These in vitro experiments suggest that Linc00969 mediates trastuzumab resistance in HER-2+ BC cells. Additionally, Linc00969 can be transported to recipient cells through exosome encapsulation and cause recipient cells to acquire trastuzumab resistance.

### Linc00969 promotes trastuzumab resistance in vivo

**Fig. 4:**
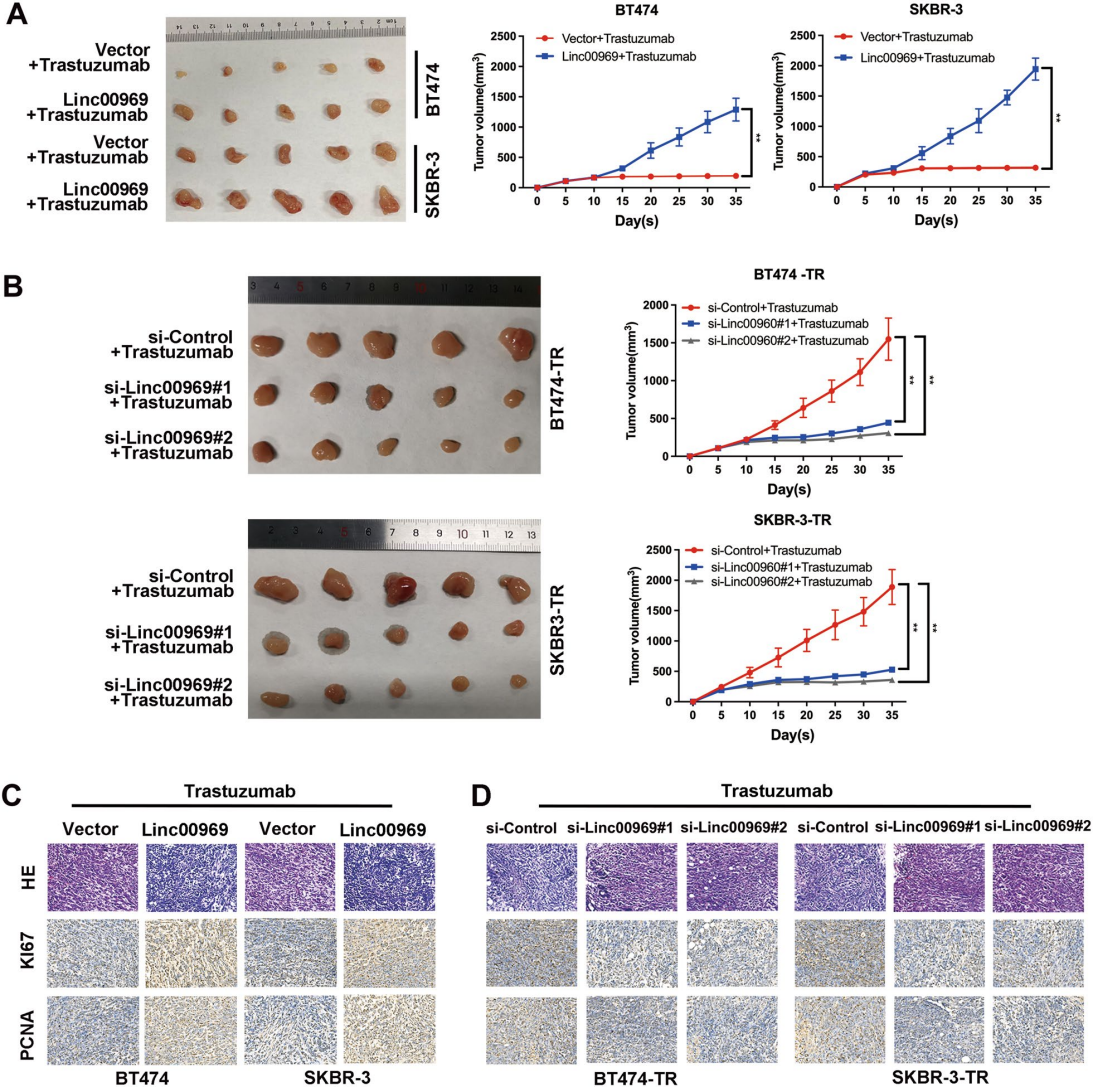
Linc00969 promotes trastuzumab resistance in breast cancer in vivo. **A** The subcutaneously transplanted tumor volumes after sacrificing nude mice and growth curves of subcutaneously transplanted tumors after injecting BT474 and SKBR-3 cells with or without the Linc00969 plasmid in the presence of trastuzumab treatment. **P < 0.01. **B** The subcutaneously transplanted tumor volumes after sacrificing nude mice and growth curves of subcutaneously transplanted tumors after injecting BT474-TR and SKBR-3-TR cells with or without si-Linc00969 in the presence of trastuzumab treatment. **P < 0.01. **C** HE staining and immunohistochemical staining for the Ki67 and PCNA proteins in subcutaneously transplanted tumors of BT474 and SKBR-3 cells with or without Linc00969 overexpression. **D** HE staining and immunohistochemical staining for the Ki67 and PCNA proteins in subcutaneously transplanted tumors of BT474-TR and SKBR-3-TR cells with or without Linc00969 knockdown

Following our in vitro experiments, we also wanted to validate our observations in vivo by establishing a xenograft BALB/c nude mouse model. We found that the volumes of subcutaneously transplanted tumors were significantly higher in the Linc00969 overexpression groups than in the regular groups after trastuzumab treatment (Fig. 4A), while the si-Linc00969 groups were more sensitive to trastuzumab than the control groups (Fig. 4B).

Then, we performed IHC staining on a mouse model to evaluate the expression levels of Ki67 and PCNA, which represented the proliferation capacity of the tumors. The expression levels of Ki67 and PCNA were markedly decreased by silencing Linc00969 (Fig. 4D) and significantly increased by Linc00969 overexpression (Fig. 4C). All of the above results proved that Linc00969 contributed to trastuzumab resistance and that silencing Linc00969 reversed trastuzumab resistance in HER-2+ BC in vivo.

### Linc00969 enhances trastuzumab resistance by upregulating HER-2 expression

**Fig. 5:**
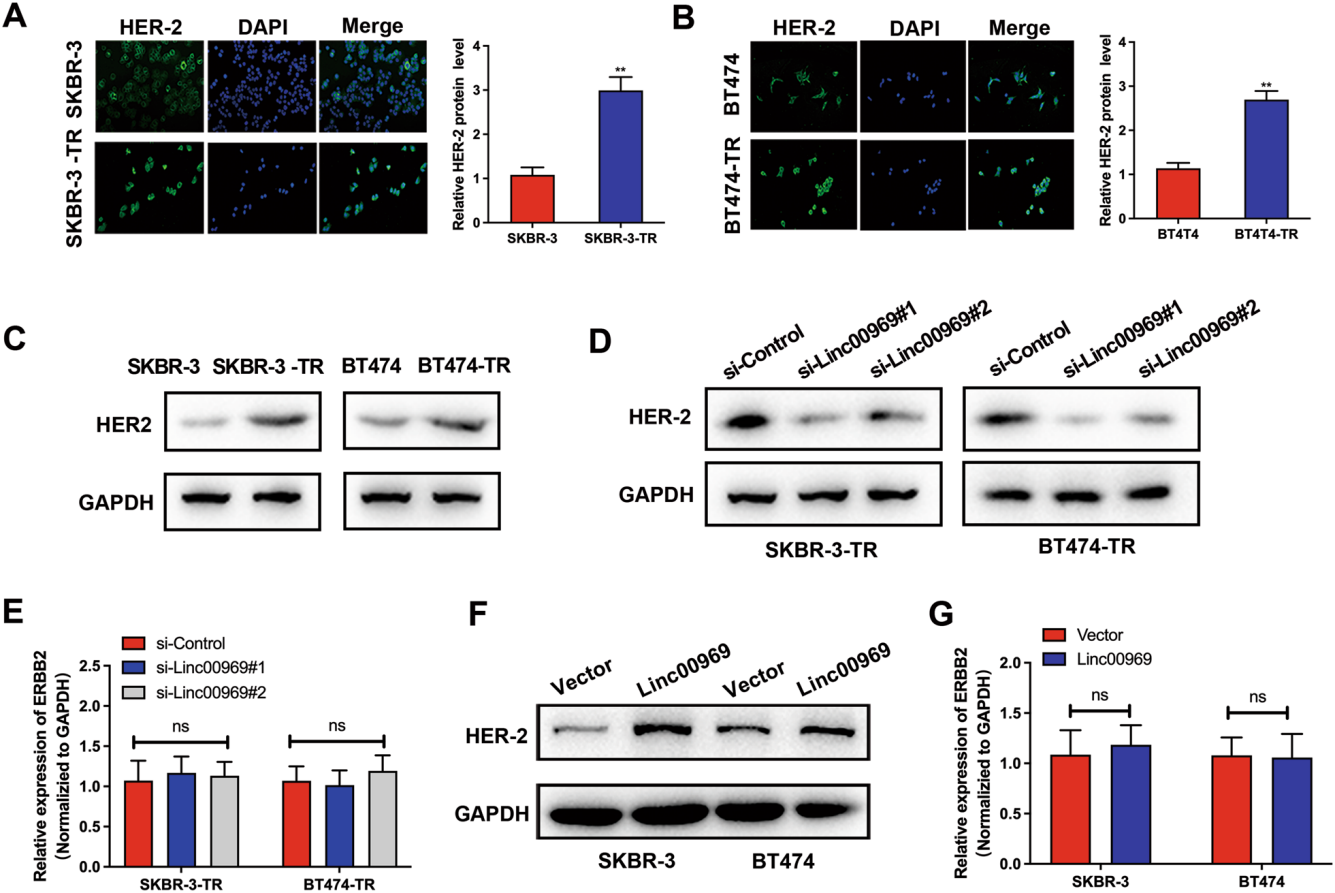
Linc00969 upregulated HER-2 expression in breast cancer cells. **A**, **B** Immunofluorescence staining showed the expression and subcellular localization of HER-2 in SKBR-3, BT474, BT474-TR and SKBR-3-TR cells. **P < 0.01. **C**, **D** HER-2 protein expression levels were measured by western blotting after silencing Linc00969. **E** The RNA expression levels of HER-2 were measured by PCR after silencing Linc00969. ns: no significance. **F** The HER-2 protein expression levels were measured by western blotting after overexpressing Linc00969. **G** The RNA expression levels of HER-2 were measured by PCR after overexpressing Linc00969. ns: no significance

Next, we explored how Linc00969 induced trastuzumab resistance. Since Linc00969 was overexpressed in HER-2+ BC cells, we first investigated the relationship between Linc00969 and HER-2 expression. Based on the results of immunofluorescence staining, we found that the HER-2 protein was mainly located in the nucleus of BC cells and upregulated in the resistant subgroups compared to the parental BC cell lines (Fig. 5A–C). Then, we found that HER-2 expression was sharply decreased by silencing Linc00969 (Fig. 5D), while overexpressing Linc00969 upregulated HER-2 protein levels (Fig. 5F). However, the expression of ERBB2 (ERBB2 indicated the coding RNA of HER-2 protein) measured by qRT‒PCR did not change when Linc00969 was silenced or overexpressed (Fig. 5E, G). Together, these results suggested that Linc00969 regulates trastuzumab resistance in HER-2+ BC cells through HER-2 expression only at the protein level but not at the transcription level.

### Linc00969 maintains HER-2 mRNA stability by binding to Hu antigen R (HUR)

**Fig. 6:**
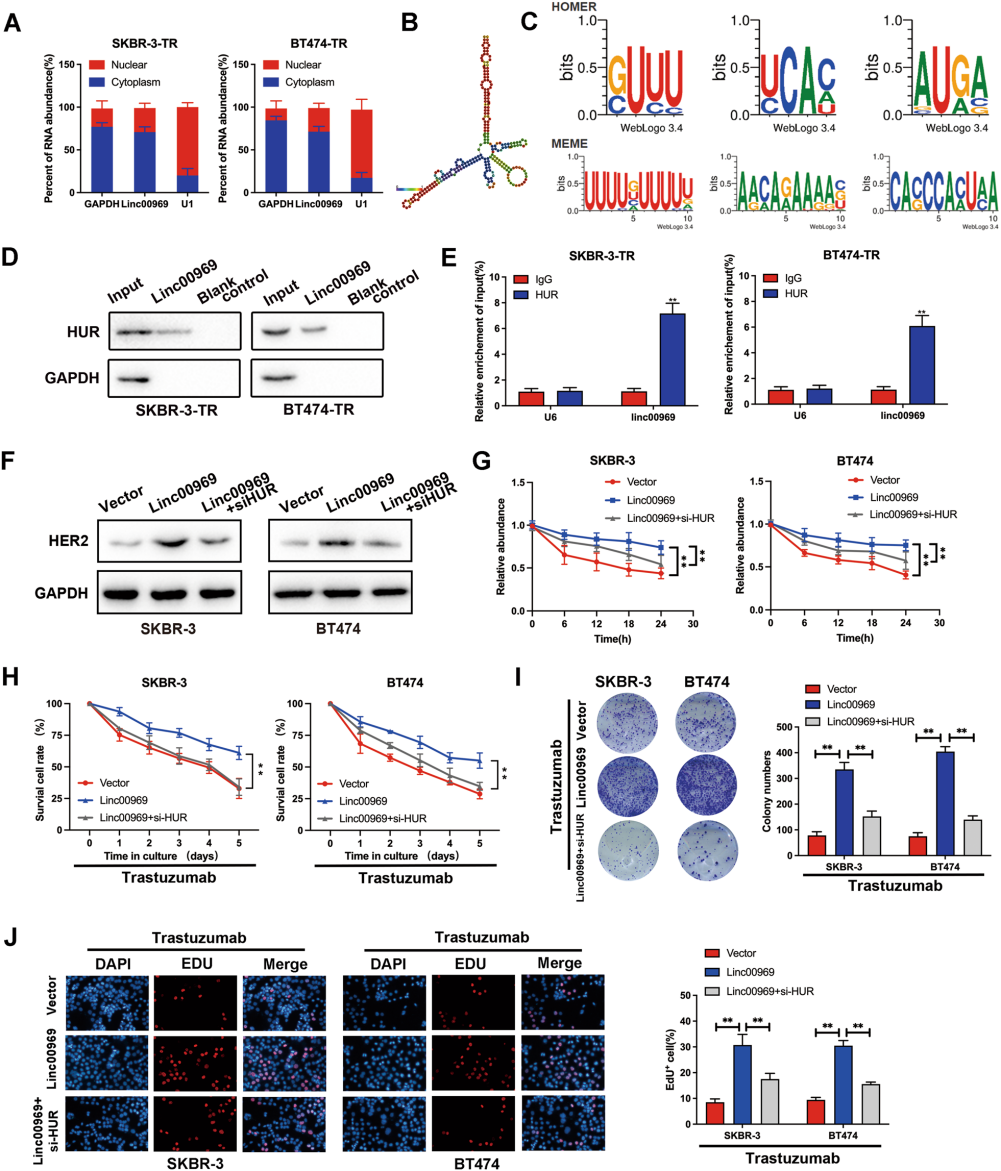
Linc00969 maintains HER-2 mRNA stability by binding to HUR. **A** Subcellular localization of Linc00969 in BT474-TR and SKBR-3-TR cells was determined by nucleus-cytoplasm fraction qPCR. **B** The secondary structure of Linc00969 predicted by online minimum free energy (MFE) evaluation (http://rna.tbi.univie.ac.at/). **C** POSTAR3 predicted that the chr3:195,689,368–195,689,390 nt region of Linc00969 contained a binding motif for HUR. **D** RNA pull-down assay showed that the HUR protein was enriched by Linc00969 in breast cancer cells. **E** RIP assay confirmed the binding relationship between HUR and Linc00969. **P < 0.01. **F** The HER-2 protein expression levels were measured by western blotting after overexpressing Linc00969 and silencing HUR in BT47 and SKBR-3 cells. **G** The stability of HER-2 mRNA was analyzed by PCR after overexpressing Linc00969 and silencing HUR in BT47 and SKBR-3 cells. **P < 0.01. **H** The CCK8 assay. **I** The colony forming assay. **J** The EdU assay of BT47 and SKBR-3 cells to trastuzumab after overexpressing Linc00969 and silencing HUR. **P < 0.01

However, the regulatory mechanism of Linc00969 in the upregulation of HER-2 expression is still unknown and needs further study. The subcellular location of Linc00969 was identified by nucleus-cytoplasm fraction qPCR, and the results demonstrated that Linc00969 was mainly located in the cytoplasm (Fig. 6A). According to the evaluation of the online minimum free energy (MFE) (http://rna.tbi.univie.ac.at/), we predicted that the Linc00969 transcript at the 1058–1305 nt locus formed a stem‒loop structure (Fig. 6B), which is the key structure related to the targeted RNA-binding proteins. It has been reported that the upregulation of HER-2 expression may occur due to HUR, the RNA-binding protein of Linc00969, which increases HER-2 mRNA stability and expression in hepatocellular carcinoma [29]. Moreover, prediction by POSTAR3 showed that the chr3:195,689,368–195,689,390 nt region of Linc00969 contained a binding motif for HUR (Fig. 6C), which indicated that Linc00969 could be a scaffold that mediates the interaction of HER-2 and HUR. Consistently, immunofluorescence assays showed that Linc00969 and HUR were colocalized mostly in the cytoplasm (Additional file 3: Fig. S3C). These results suggest that Linc00969 may interact with HUR proteins. Furthermore, we designed a Linc00969 probe, performed an RNA pull-down assay, and found that the HUR protein was enriched by Linc00969 (Fig. 6D). RIP assays were also performed to prove the direct interaction between Linc00969 and HUR (Fig. 6E). However, HUR was not affected by Linc00969 knockdown at the transcriptional level **(**Additional file 3: Fig. S3D). Silencing HUR with si-HUR (Additional file 3: Fig. S3A, B) significantly abrogated the Linc00969-induced increase in HER-2 protein (Fig. 6F). Moreover, overexpression of Linc00969 increased the stability of HER2 mRNA; however, this effect was significantly reversed in HUR-knockdown cells (Fig. 6G), indicating that HUR was essential for Linc00969-induced HER-2 mRNA stability. CCK8, colony formation and EdU assays showed that the trastuzumab resistance effect after overexpression of Linc00969 could be partially restored by blocking HUR in both SKBR-3 and BT474 cells (Fig. 6H–J). The above results indicated that Linc00969 promoted trastuzumab resistance in HER-2+ BC cells by maintaining HER-2 mRNA stability by binding to HUR.

### Exosomal Linc00969 regulates trastuzumab resistance by inducing autophagy

**Fig. 7:**
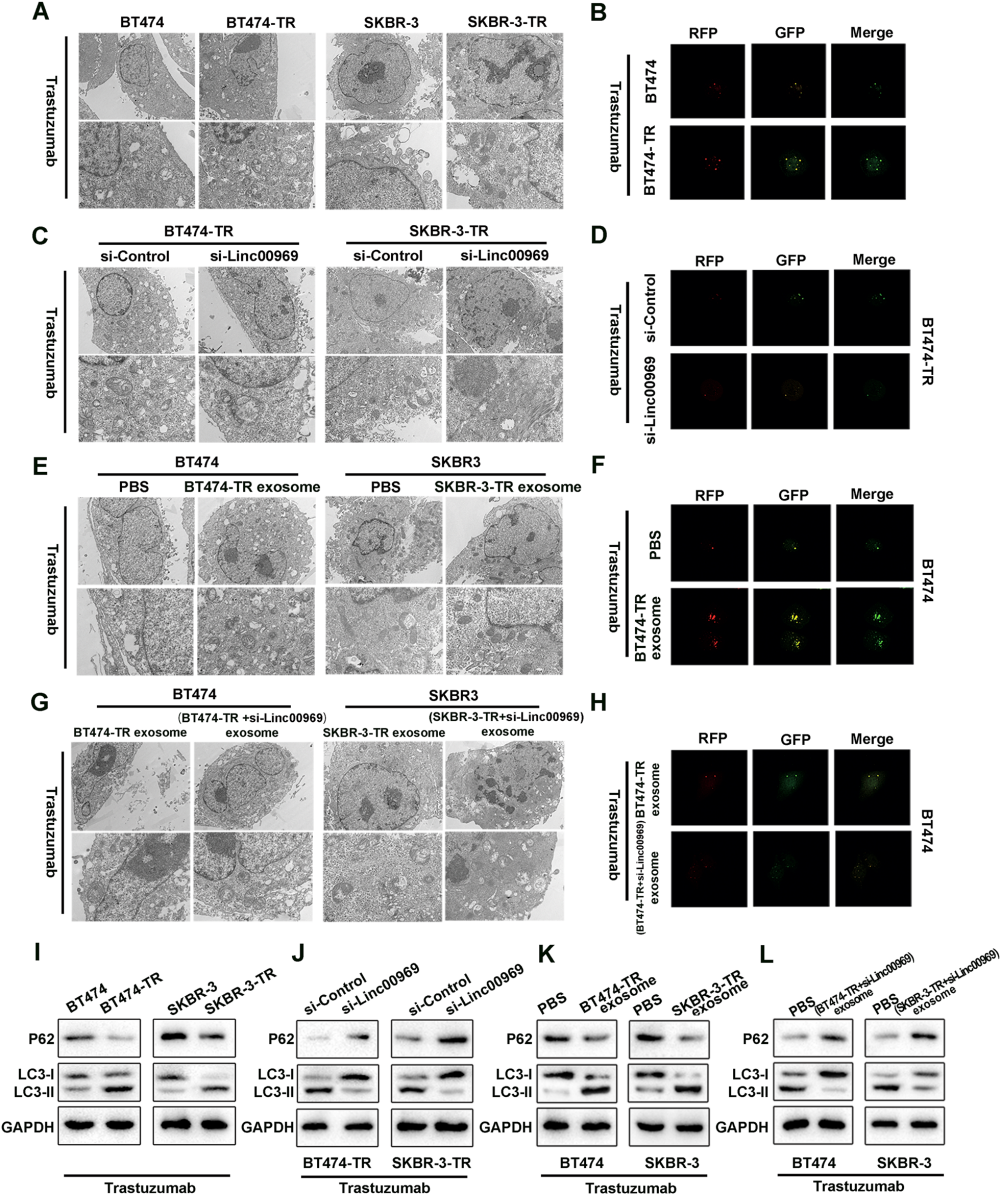
Exosomal Linc00969 promotes trastuzumab resistance in breast cancer by inducing autophagy. **A**, **B** Autophagosomes were observed by scanning electron microscopy (SEM) and confocal laser scanning fluorescence microscopy in BT474, SKBR-3, BT474-TR and SKBR-3-TR cells after trastuzumab treatment. **C**, **D** Autophagosomes were observed by SEM and confocal laser scanning fluorescence microscopy in BT474-TR and SKBR-3-TR cells with or without si-Linc00969 after trastuzumab treatment. **E**, **F** Autophagosomes in BT474 and SKBR-3 cells after trastuzumab treatment with or without exosomes from trastuzumab-resistant cells. **G**, **H** Autophagosomes in BT474 and SKBR-3 cells after trastuzumab treatment with exosomes from respective trastuzumab-resistant cells with or without Linc00969 knockdown. **I**, **L** The p62 and LC3 protein levels were measured by western blotting in breast cancer cells after trastuzumab treatment

Autophagy might induce drug resistance by decreasing the cytotoxicity of drugs to cancer cells. To investigate whether exosomal Linc00969 regulates the resistance of trastuzumab via autophagy, we analyzed the level of autophagy in BC cells. We found that the trastuzumab-resistant BC cells showed markedly higher levels of autophagy than the parental cells, as evidenced by increased formation of autophagosomes (Fig. 7A), increased autophagic flux (Fig. 7B), increased LC3-II (Light Chain-3) expression (the biomarker of autophagy) and decreased expression of p62 protein (biomarker of autophagy suppression) (Fig. 7I), confirming the close relationship between trastuzumab resistance and autophagy in BC cells. Then, we explored whether Linc00969 influences the expression level of autophagy in BC cells. In contrast to the control group, silencing Linc00969 reduced the number of autophagosomes (Fig. 7C), decreased the autophagic flux (Fig. 7D), decreased LC3-II expression and elevated p62 expression (Fig. 7J) in the presence of trastuzumab treatment. More importantly, parental BC cells cultured with exosomes derived from trastuzumab-resistant cells exhibited increased autophagy activity (Fig. 7E, F, K). However, silencing Linc00969 sharply inhibited the ability of cocultured BC cells to increase autophagy activity (Fig. 7G, H, L). Furthermore, we found that inhibition of autophagy with HCQ can reverse linc00969 trastuzumab resistance (Additional file 4: Fig. S4). In summary, we confirmed that exosomal Linc00969 could transmit trastuzumab resistance by regulating autophagy levels.

## Discussion

Breast cancer is the most common malignant disease in female patients worldwide [1]. Breast cancer can be subtyped by gene expression profiling, and these subtypes include basal-like, triple-negative (TN) nonbasal, human epithelial growth factor receptor 2 (HER-2) enriched, luminal A, luminal B and luminal/HER-2 breast cancer [30]. HER-2+ breast cancer patients who were treated with a combination of systemic therapy and anti-HER-2 therapy (trastuzumab) had longer overall survival (OS) and progression-free survival (PFS) than those who received only systemic therapy [31]. However, approximately one-third of HER-2+ BC patients experience recurrence or metastasis because of trastuzumab resistance [2, 5]. Therefore, an understanding of the mechanism of trastuzumab resistance is important for developing effective and novel therapies to treat HER-2+ BC patients.

In previous studies, some researchers have mentioned that exosomes and lncRNAs are associated with tumor drug resistance [20–22] and are probably related to trastuzumab resistance [24–26]. However, there is no direct evidence to prove the role and mechanism of exosomal lncRNAs in trastuzumab resistance in HER-2+ BC. Therefore, we collected and compared the plasma exosomes of patients with and without trastuzumab resistance and identified the lncRNA Linc00969, which was overexpressed in HER-2+ BC patients with trastuzumab resistance. Then, we established the trastuzumab-resistant BC cell lines SKBR-3-TR and BT474-TR to clarify the role and mechanism of exosomal lncRNA Linc00969 in trastuzumab resistance in BC in vitro and in vivo.

According to our results, we first successfully isolated and identified exosomes derived from BC patients and cells. We found that exosomes secreted from BC cells with trastuzumab resistance could be endocytosed by parental BC cells and enhance the resistance of BC cells to trastuzumab. Second, we identified that Linc00969 was overexpressed in the exosomes of trastuzumab-resistant BC patients through microarray profiling and validated it by qPCR assay in 108 BC patients, including early-stage and metastatic BC patients. We also confirmed that positive nodal status, higher histological grade, higher Ki67 score and distant metastasis were correlated with higher exosome Linc00969 expression in BC patients. Furthermore, we proved that Linc00696 was encapsulated by exosomes and overexpressed in trastuzumab-resistant BC cells. If we used siRNA to knockdown exosomal lncRNA Linc00969, the trastuzumab resistance of SKBR-3-TR and BT474-TR cells was decreased in vitro and in vivo, and the trastuzumab resistance of BT474 and SKBR-3 cells could be enhanced by overexpressing Linc00696 in vitro and in vivo. Thus, we have proven that exosomal lncRNA Linc00969 is correlated with trastuzumab resistance in BC. Furthermore, we found that Linc00969 could regulate trastuzumab resistance by promoting HER-2 expression at the protein level. However, it is not well understood how Linc00969 plays a role in regulating HER2 expression, which is involved in trastuzumab resistance.

The RNA-binding protein Hu antigen R (HUR) can act as a posttranscriptional regulator. The expression levels of HUR are regulated by a variety of proteins, microRNAs and so on [32]. HUR is upregulated in BC and is involved in the stability of various mRNAs and the translation of genes associated with breast cancer formation, metastasis, progression and therapy [32, 33]. HUR is considered an oncogenic protein that is related to more aggressive forms of BC and poor clinical outcomes [33–35]. Currently, an increasing number of researchers consider HUR to be a critical drug target in BC treatment. Wu et al. reported that the HUR inhibitor KH-3 could suppress the growth and invasion of BC in vitro and in vivo by disrupting the HuR-FOXQ1 mRNA interaction [33]. HUR, as an RNA binding protein, can also mediate the upregulation of mRNA stability, such as binding and stabilizing HSPD1 to promote the proliferation and metastasis of BC [36]. Moreover, HUR can stabilize the mRNA of CPT1 and enhance drug resistance in trastuzumab-resistant BC [37]. Furthermore, HUR can function as the RNA binding protein of HER-2 that mediates its mRNA stability and upregulates its expression in hepatocellular carcinoma [29]. Therefore, we further investigated whether HUR can bind to HER-2 mRNA to regulate its stability and expression in trastuzumab-resistant BC based on the above studies. In our study, we found that HUR was mainly located in the nucleus of trastuzumab-resistant BC cells, and the pull-down assay and RIP assay showed the interaction between Linc00969 and the HUR protein. Then, we proved that Linc00969 could increase HER-2 protein expression and enhance the stability of HER-2 mRNA by binding to HUR, which enhances trastuzumab resistance in BC.

Autophagy is a very complicated process in which double membrane vesicles named autophagosomes are formed. It maintains cellular homeostasis by degrading intracellular molecules and organelles [38]. Autophagy plays a very important role in developing and differentiating hollow lumen structures and maintaining homeostasis in normal mammary tissue [39]. In BC, autophagy can protect normal mammary cells from various intrinsic and extrinsic stresses, which can cause instability of and mutations in DNA and finally lead to the formation of preneoplasm and hyperproliferation [40]. However, targeting autophagy is complicated because autophagy is a process that exerts both death-inducing and survival-promoting effects in BC [41]. Several studies have highlighted autophagy as a mechanism for trastuzumab resistance in HER-2+ BC [41, 42]. These studies suggest that trastuzumab sensitivity could be enhanced by inhibiting autophagy [43]. However, no studies have shown that lncRNAs can enhance trastuzumab resistance by inducing autophagy. In our study, we also found that the number of autophagosomes and the level of LC3-II protein were much higher in BC cells with trastuzumab resistance. When we blocked Linc00969 expression, the formation of autophagosomes and LC3-II protein level were decreased in SKBR-3-TR and BT474-TR BC cells. The autophagosomes formation and LC3-II expression in parental BC cells were increased when we added exosomes from trastuzumab-resistant cells. Our results first suggested that exosomal lncRNA Linc00969 might also be associated with trastuzumab resistance in BC by inducing autophagy.

## Conclusion

In conclusion, trastuzumab resistance, either de novo or acquired, is an important clinical challenge in the treatment of BC patients. Currently, HER-2 amplification status alone cannot explain the mechanisms underlying disease progression and drug resistance [44]. To improve BC patient outcomes, it is urgent to recognize and understand the underlying pathway and mechanisms involved in trastuzumab resistance. In this study, we first found and suggested that exosomal lncRNA Linc00969 might induce trastuzumab resistance by promoting HER-2 expression at the protein level and that Linc00969 might increase HER-2 protein expression and enhance the stability of HER-2 mRNA by interacting with HUR. Furthermore, our results also suggested for the first time that lncRNA Linc00969 might also be associated with trastuzumab resistance by inducing autophagy in BC. Our results possibly indicate a novel mechanism underlying trastuzumab resistance and are clinically relevant for improving the treatments and outcomes of HER-2+ BC patients.

### Supplementary Information

**Additional file 1: Fig. S1**. The isolation and identification of exosomes. (A) The morphology and size of isolated exosomes from trastuzumab-resistant breast cancer (BC) patients (R-exo) and trastuzumab-sensitive BC patients (S-exo) were observed by electron microscopy. (B) The biomarkers of exosomes from BC patients were detected by western blotting. (C) The size and number of exosomes from BC patients. (D) The morphology and size of exosomes from SKBR-3 and SKBR-3-TR cells were observed by electron microscopy. (E) The biomarkers of exosomes from BC cells were detected by western blotting. (F) The particle size and number identification of isolated exosomes from BC cells. (G) The survival rates of BC cells in response to trastuzumab were determined by CCK8 assay. **P<0.01. (H) qPCR assays validated the overexpressed levels of Linc00969 in tissue samples from trastuzumab-resistant BC patients (R-exo) compared with plasma exosomes from trastuzumab-sensitive BC patients (S-exo). ***P < 0.001

**Additional file 2: Fig. S2**. Trastuzumab resistance was increased in breast cancer cells after Linc00969 overexpression. (A) The RNA expression levels of Linc00969 and exosomal Linc00969 in breast cancer (BC) cells with or without si-Linc00969. **P<0.01. (B) The RNA expression levels of Linc00969 and exosomal Linc00969 after transfection of the Linc00969 plasmid into BC cells. **P<0.01. (C) CCK8 assay. (D) Colony forming assay. (E) EdU assay of BC cells treated with trastuzumab after overexpression of Linc00969. **P<0.01.

**Additional file 3: Fig. S3**. Silencing HUR in trastuzumab-resistant breast cancer cells. (A) The RNA expression levels of HUR measured by PCR in trastuzumab-resistant breast cancer (BC) cells after treatment with si-HUR. **P<0.01. (B) The HUR protein expression levels were measured by western blotting after silencing HUR in trastuzumab-resistant BC cells. (C) Immunofluorescence staining showed the subcellular localization of HUR in trastuzumab-resistant BC cells. (D) The mRNA expression levels of HUR measured by PCR in trastuzumab-resistant BC cells after silencing Linc00969. ns: no significance.

**Additional file 4: Fig. S4**. Trastuzumab resistance was reversed in breast cancer cells after inhibition of autophagy with HCQ. (A) CCK8 assay. (B) Colony forming assay. (C) EdU assay of BC cells treated with trastuzumab after inhibition of autophagy with HCQ. **P<0.01.

**Additional file 5: Table S1**. The lncRNAs that were differentially expressed in exosomes from trastuzumab-sensitive breast cancer tissues compared with trastuzumab-resistant breast cancer tissues.

**Additional file 6: Table S2**. The CirRNAs that were differentially expressed in exosomes from trastuzumab-sensitive breast cancer tissues compared with trastuzumab-resistant breast cancer tissues.

**Additional file 7: Table S3**. The correlation between exosomal Linc00969 expression and clinicopathological features of 108 breast cancer patients.

## Data Availability

The datasets analyzed during the current study are not publicly available, but are available from the corresponding author on reasonable request.

## Abbreviations

BC: Breast cancer
HER-2: Human epithelial growth factor receptor 2
ATCC: American type culture collection
CCK8: Cell Counting Kit-8
EdU: 5-Ethynyl-2-deoxyuridine
PVDF: Polyvinylidene fluoride
DAPI: 4′,6-Diamidino-2-phenylindole
FISH: Fluorescence probe in situ hybridization
RIP: RNA binding protein immunoprecipitation
DERs: Differentially expressed RNAs
ROC: Receiver operating characteristic
AUC: Area under curve
MFE: Minimum free energy
HUR: Hu antigen R
LC3: Light chain-3
TN: Triple-negative
OS: Overall survival
PFS: Progression-free survival
