# Supplementary material for: Exosomal Linc00969 induces trastuzumab resistance in breast cancer by increasing HER-2 protein expression and mRNA stability by binding to HUR
Source: Breast Cancer Res. 2023 Oct 17;25:124. doi: 10.1186/s13058-023-01720-6 (PMC10580635; doi:10.1186/s13058-023-01720-6)
Supplement: Supplementary file 7 — Additional file 7: Table S3. The correlation between exosomal Linc00969 expression and clinicopathological features of 108 breast cancer patients. [file 13058_2023_1720_MOESM7_ESM.docx]

Supplementary Table 2. Correlation between exosome Linc00969 expression and clinicopathological features in 108BCa patients exosomes

| Characteristics | case number | linc00969 | | P value |
| --- | --- | --- | --- | --- |
|  |  | low | high |  |
| All cases | 108 | 54 | 54 |  |
| Age (years) |  |  |  | 0.286 |
| < 50 | 56 | 29 | 27 |  |
| ≥50 | 52 | 25 | 27 |  |
| Menopausal status |  |  |  | 0.498 |
| Pre | 63 | 27 | 36 |  |
| Post | 45 | 27 | 18 |  |
| Clinical tumor size |  |  |  | 0.438 |
| T1 | 22 | 9 | 13 |  |
| T2-4 | 86 | 45 | 41 |  |
| Clinical nodal status |  |  |  | <0.001*** |
| N0 | 30 | 22 | 8 |  |
| N+ | 78 | 32 | 46 |  |
| Histological grade |  |  |  | 0.033* |
| 1 | 6 | 4 | 2 |  |
| 2/3 | 102 | 50 | 52 |  |
| Ki67 score (%) |  |  |  | 0.011* |
| <40 | 48 | 26 | 22 |  |
| ≥40 | 60 | 28 | 32 |  |
| HR status |  |  |  | 0.117 |
| Positive | 31 | 11 | 20 |  |
| Negative | 77 | 43 | 34 |  |
| Distant metastasis |  |  |  | 0.002** |
| No | 44 | 28 | 16 |  |
| Yes | 64 | 26 | 38 |  |
| Objective response |  |  |  | <0.001*** |
| Complete | 4 | 3 | 1 |  |
| Partial | 58 | 37 | 21 |  |
| Progressive disease | 46 | 4 | 42 |  |

* *P*<0.05, ** *P*<0.01, *** *P*<0.001
